# Slip Risk on Surfaces Made with 3D Printing Technology

**DOI:** 10.3390/ma18030573

**Published:** 2025-01-27

**Authors:** Bartosz Wieczorek, Łukasz Gierz, Łukasz Warguła, Grzegorz Kinal, Boris Kostov, Konrd Jan Waluś

**Affiliations:** 1 Faculty of Mechanical Engineering, Institute of Machine Design, Poznan University of Technology, Piotrowo 3 Street, 60-965 Poznan, Poland; bartosz.wieczorek@put.poznan.pl (B.W.); lukasz.wargula@put.poznan.pl (Ł.W.); konrad.walus@put.poznan.pl (K.J.W.); 2 Faculty of Civil and Transport Engineering, Institute of Machines and Motor Vehicles, Poznan University of Technology, Piotrowo 3 Street, 60-965 Poznan, Poland; grzegorz.kinal@put.poznan.pl; 3 Department of Heat, Hydraulics and Environmental Engineering, University of Rousse “Angel Kanchev”, Studentska 8 Street, 7017 Ruse, Bulgaria; bkostov@uni-ruse.bg

**Keywords:** British Portable Skid Resistance Tester (BSRT), Slip Resistance Value (SRV), Polylactic Acid (PLA), Polyethylene Terephthalate Glycol (PET-G), Thermoplastic Polyurethane (TPU), surface roughness, 3D printing process parameters

## Abstract

Slip risk on surfaces used by humans or active in mechanisms is studied to mitigate its effects or harness its beneficial outcomes. This article presents pioneering research on the risk of surfaces created using 3D printing technology. The study examines three materials (Polylactic Acid, PLA; Polyethylene Terephthalate Glycol, PET-G; and Thermoplastic Polyurethane, TPU), considering three print head movement directions relative to the British Portable Skid Resistance Tester (BSRT) measurement direction. In addition, surface roughness tests were performed. Dry tests showed that the structure created by the printing direction perpendicular to the movement direction is the safest in terms of slip risk. The SRVs of the measured samples on a qualitative scale were classified on this scale as materials with low or extremely low slip risk (ranging from 55 to 90 SRV dry and 35 to 60 SRV wet). Referring to the influence of the type of material on the SRV, it was found that the safest material in terms of reducing the risk of slipping in dry conditions is TPU and, in wet conditions, PLA. During wet tests, the best properties that reduce the risk of slippage in most cases are shown by the printing direction on a horizontal plane at an angle of 45° to the direction of movement. Statistical analysis showed that the printing direction and roughness do not have a statistically significant effect on the SRV, but the type of material and the type of method (dry and wet) and their interaction have a significant effect.

## 1. Introduction

3D printing is becoming an increasingly popular method for prototyping [1] and manufacturing parts [2]. Two examples of applications where surface slip is important. The first is the surfaces on which people and vehicles move, where the risk of slippage poses a threat to life and health [3,4]. The second application involves slip in transmission systems with pulleys and V-belts or flat belts, where belt slip can function as an overload clutch [5,6] and affect the efficiency of the gear system and the energy consumption of the entire transport process [7]. This is particularly important when prototyping new concepts of non-circular wheels with complex shapes.

Slip risk on surfaces created by 3D printing is a topic of importance from both user safety [8] and surface engineering perspectives [9]. In the scientific literature, this issue is examined in terms of surface microstructure [10] and the mechanical [11] and chemical [12] properties of 3D printed materials. Surfaces made with technologies such as FDM (Fused Deposition Modeling) or SLA (Stereolithography) frequently display uneven textures due to the layered deposition process [13,14,15], resulting in varying friction coefficients that affect the risk of slip [16,17]. Studies show that the rough surface characteristic of 3D printing can increase adhesion [18,19], but can also promote contaminant accumulation, potentially impacting user safety [20]. Commonly used 3D printing materials, such as PLA (Polylactic Acid), ABS (acrylonitrile–butadiene–styrene), and epoxy resins, differ in mechanical and chemical properties [21,22,23], influencing surface slip characteristics. For example, hydrophilic properties [24] can increase the risk under humid conditions [25]. Various post-processing methods, such as sanding [26], polishing [27,28], and coating [29], can adjust the friction coefficient of 3D printed surfaces. Research indicates that anti-slip [30] or hydrophobic [31] coatings can significantly reduce the risk of slip and improve safety. However, inadequate procedures or improper application of coatings may lead to reduced adhesion and increased slip risk. Different surface finishes, such as glossy or matte lacquer [4], can also contribute to the risk of slip.

In mechanical applications, the slip risk between a V-belt or flat belt and a pulley printed in 3D technology is crucial, especially where the durability and performance of the drive system are key. Printed pulleys may differ from conventionally produced ones, affecting belt engagement. FDM or SLA-printed pulleys often lack the precise surface finish of traditional methods, affecting belt adherence, especially at high loads or speeds [32]. Studies highlight that the quality of 3D printed surfaces depends on parameters such as layer thickness, extruder temperature, and print speed, while material choice (eg, PLA, nylon, TPU) affects pulley-belt friction and grip [33]. The stress force on the belt directly influences the risk of slippage, with insufficient stress leading to reduced adhesion, particularly with printed pulleys prone to deformation [34]. High loads or sudden speed changes can also increase the risk if printed pulleys lack suitable hardness or reinforcement [35,36]. For V-belt pulleys, precisely designed grooves are essential for secure belt engagement, yet achieving such precision in 3D printing can be challenging due to print accuracy limitations. Grooving inaccuracies may lead to slippage, particularly if the belt does not align with the pulley profile. Post-processing of printed pulley surfaces, including silicone or synthetic rubber coatings, can significantly improve grip and belt performance by increasing friction and minimizing slip risk [37]. Noncircular pulley prototypes, though cost-effective and design-flexible, carry slip risks linked to surface quality, material choice, and production accuracy. Tailoring the print parameters, selecting suitable materials, and applying finishing techniques can effectively reduce these risks.

This article aims to conduct initial research on the risk on surfaces created using 3D printing technology. The study examines three materials (Polylactic Acid (PLA), Polyethylene Terephthalate Glycol (PET-G), and Thermoplastic Polyurethane (TPU) across three print head movement directions relative to the British Portable Skid Resistance Tester (BSRT) measurement direction. Surface roughness tests were also performed.

## 2. Materials and Methods

### 2.1. Measuring Devices

#### 2.1.1. British Portable Skid Resistance Tester

During the slip resistance test, the test was performed with the use of a British Portable Skid Resistance Tester (BSRT). During the test, the pendulum was equipped with a type 96 slider according to the AS 4586 standard [38]. This type of slider is dedicated to testing interior floors characterized by precise finishing. The British Portable Skid Resistance Tester (BSRT) was prepared for testing according to the BS EN 13036-4:2011 standard. Before the test, the slider was kept in a darkroom at a temperature close to 20 °C. The surface on which the BSRT was used had a temperature of 23 ± 2 °C. During the test, the British Pendulum Number (BPN) was read from the BSRT for 20 measurements performed in two directions of attack. Based on the obtained set of BPN values, a statistical analysis was performed according to the BS EN 13036-4:2011 standard, based on which the final result for the tested sample was determined, described as the Slip Resistance Value (SRV). The SRV was compared with a four-point qualitative scale (Table 1) recommended by the UK Slip Resistance Group [39]. This scale is in accordance with the HB 198:2014 standard [40]. If the case is that the cited qualitative scale, it is possible to use the numerical Slip Resistance Value (SRV) scale to compare the risk of slippage of materials located in the same place on the qualitative scale.

The measurement error of the British Portable Skid Resistance Tester (BSRT), used to determine the SRV, and the methods of measurement with this device depend on several factors, but are generally guided by the specifications outlined in relevant standards. The accuracy of the BSRT is in accordance with EN 13036-4:2011, which specifies a measurement error of ±0.5 SRV units. In practice, this means that the measured SRV can deviate by up to ±0.5 from the actual friction value. The error associated with the measurement method includes repeatability and reproducibility. Repeatability, defined as the variation in results obtained by the same operator using the same device under controlled conditions, is approximately ±2 SRV units. Reproducibility, which reflects the variation in results obtained by different operators using different devices, is approximately ±3 SRV units. These values are considered typical and are based on analyses provided in standards such as EN 13036-4:2011 [41] and BS 812:114 [42].

#### 2.1.2. Carl Zeiss Jena Profilometr

Measurements related to 2D surface stereometry were performed using a Carl Zeiss Jena profilometer (Jena, Germany), (type—contact, model—ME-10) equipped with heads with an inductive transducer and SUFORM software from SAJD METROLOGIA, which modernized the profilograph meter in 2000. A measuring arm with a diamond contact tip (without a slider) in the shape of a pyramid with a 2 μm top rounding was used in the measurements. The measuring head of the device can move in two perpendicular axes, while the table on which the samples rested allows for movement in the horizontal plane. The vertical range during measurements when using the inductive head was ±200 μm. The movement of the measuring needle allows for the performance of a profilogram with a maximum length of up to 100 mm (profilograms were performed in the tests where the maximum length did not exceed 15 mm). The applied travel speed of the measuring tip during the measurements was 0.1 mm/s (the device capabilities are 0.01 mm/s to 1 mm/s).

### 2.2. Measurement Procedures

#### 2.2.1. British Pendulum Measurement Procedure

The measurement procedure assumed 8 repetitions of the slip test from two opposite directions of attack. Slip tests were performed with a pendulum arm equipped with a 96-type slider. The tests were carried out dry and wet. During the wet measurement, the sample was sprayed with water at room temperature immediately before slip test. This measurement procedure and the slider selected in this way met the definition of the risk of sliding on internal surfaces. According to the BS EN 13036-4:2011 standard and the manufacturer’s guidelines for the pendulum used (The Munro Stanley Portable Skid Resistance Tester) [41]. To determine the SRV, a total of 18 BPN measurements were performed for each sample, of which 15% of the extreme BPN results were rejected. For the remaining set of values, an average was calculated, which was the SRV allowing a comparative analysis of the material samples located in the same place on the quality scale. Statistical analysis of the results SRVs of the presented used Student’s t-distribution, based on which the confidence interval was determined for the probability level *p* = 0.05.

#### 2.2.2. Roughness Measurement Procedure

**Figure 1:**
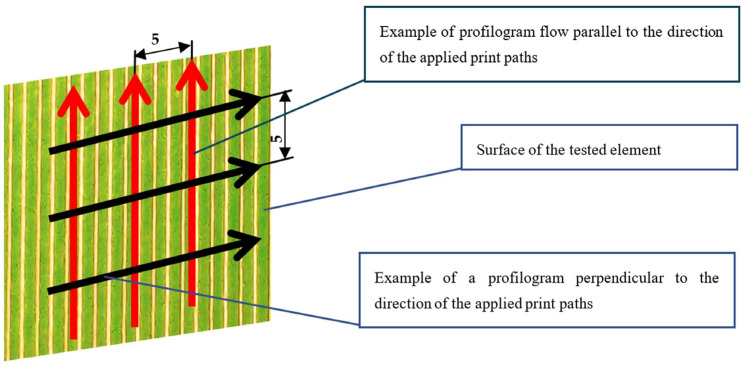
Schematic representation of the profilogram locations perpendicular and parallel to the applied layers on the surface of the tested samples.

The measurement procedure was carried out setting the elementary section λc = 2.5 mm, where the corresponding measurement section Ln was 10 mm. The length of the elementary section was determined based on the dependence of the elementary section length value on the expected values of the roughness parameters Ra according to the standards of PN-EN ISO 3274:1997 [43] and PN-EN ISO 4288:1997 [44] standards. Taking into account each prepared sample, six profilograms were made, three of which were made perpendicular to the applied print paths and the next three were made parallel to the applied print paths. The location of the measurements for a given plate was randomly selected. The distances between the individual profilograms, made parallel and perpendicular to the direction of the applied print paths, were 5 mm. The schematic location of the profilograms on the surface of the tested samples (elements) is shown in Figure 1.

For each of the profilograms performed, the Ra parameter was determined (being the arithmetic mean of the profile ordinate values) characterizing the surface roughness of the tested samples in randomly selected places in an area of approximately 100 mm^2^ (taking into account the distribution of individual profilograms). During the analysis of each profilogram, cutoff filtration was used. During the analysis of the test results, the terminology compliant with the standards PN-M-04250:1987 [45] and PN-EN ISO 4287:1999 [46] was used.

During the analysis of the measurement results, the arithmetic mean of a given set of results was determined and the dispersion of the measurement results of a given parameter was determined by determining the limits of the confidence intervals for the mean value at the significance level of α = 0.1.

**Figure 2:**
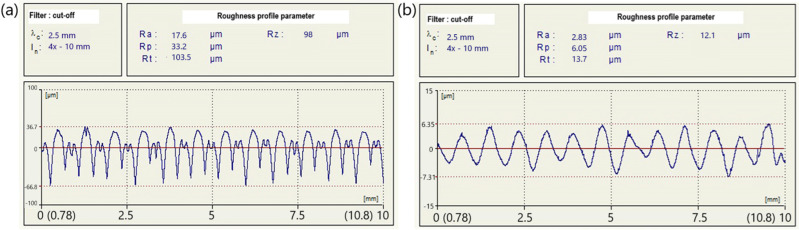
Examples of surface roughness profiles obtained for the TPU material with the PD 90° printing path direction and the profilograms (**a**,**b**) parallel to the direction of the applied printing paths.

The measurement results of the tested roughness parameter Ra were obtained based on the profiles of the tested surface taken at a specific location on the sample (the location was randomly selected in the middle of the sample as the most representative). Examples of roughness profiles for the TPU material for the PD 90° path direction over a distance of 10 mm, together with the determined tested roughness parameter, are shown in Figure 2.

### 2.3. Characteristics of the Research Plates and Materials Used

**Figure 3:**
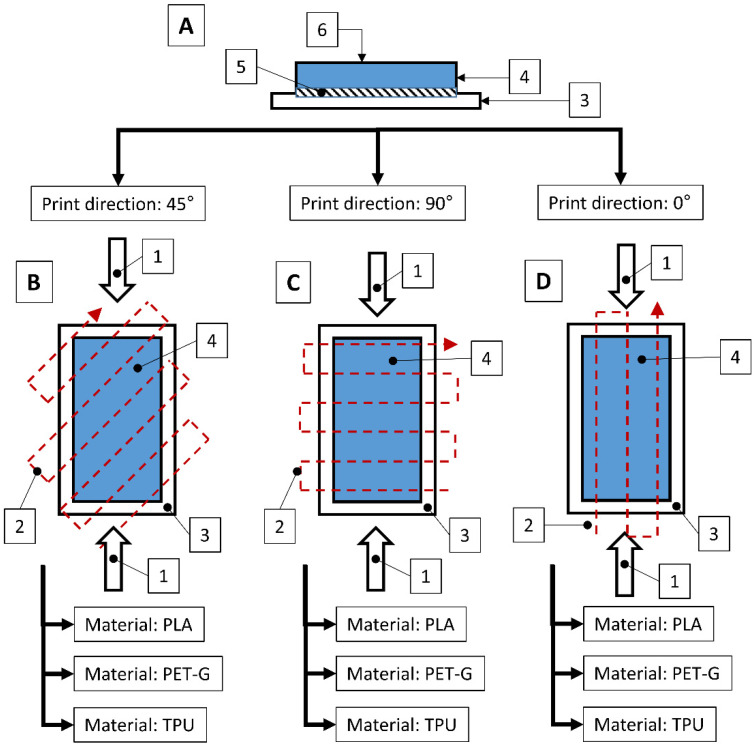
Structure of the tested samples with a detailed design of the prepared samples, directions of the print head movement relative to the BPN measurement direction, and material variants. Where A—diagram of the construction of the sample placed in the measuring apparatus, B—measure sample with the print path at an angle of 45° relative to the measurement direction., C—measuring sample with the print path at an angle of 90° relative to the measurement direction, D—measuring sample with the print path at an angle of 0° relative to the measurement direction, 1—direction of pendulum movement, 2—direction of the head movement during sample printing, 3—rigid stabilizing plate, 4—sample made using 3D printing technology, 5—cyanoacrylate adhesive layer, 6—tested surface.

The tests were performed on samples (Figure 3) made using 3D printing technology from three different materials: Polylactic Acid (PLA), Polyethylene Terephthalate Glycol (PET-G), and Thermoplastic Polyurethane (TPU). An additional variable parameter was the direction of the print head path relative to the adopted direction of roughness measurement and slip risk. According to these variables, 9 samples were used in the test. Each sample was glued with cyanoacrylate glue to a rigid stabilizing plate that prevented deformations affecting the flatness of the tested surface.

In order to more accurately present the tested samples (9 pcs.) of the produced wafers, photos were taken with a Bresser BIOLUX ICD BINO 80× LED optical microscope with a 2 MP CMOS camera, which are presented in Table 2. Photos were taken at 30× magnification to highlight the paths applied by the Ender-3 V2 3 D printer, Creality, Shenzhen, China. The printer was in a closed room with a constant temperature of 25 °C during the preparation of the printed boards. Detailed printing parameters for the three specified materials (PLA, PET-G, TPU) are listed in Table 3. These parameters were selected according to the guidelines of the manufacturers of the materials used. The printed rectangular-shaped test plates had dimensions of 100 × 150 mm^2^ and a thickness of 4 mm.

The molecular characteristics of the tested materials are as follows:

- Polylactic Acid (PLA): PLA is a crystalline material with good rigidity and low flexibility. It has a molecular weight (average weight, Mw) of approximately 80,000–100,000 g/mol and a glass transition temperature of 55–60 °C.
- Polyethylene Terephthalate Glycol (PET-G): PET-G is an amorphous polymer known for its higher chemical resistance and greater flexibility compared to PLA. The molecular weight of PET-G is approximately 30,000–40,000 g/mol, and its glass transition temperature is around 70–80 °C.
- Thermoplastic Polyurethane (TPU): TPU is a flexible material with excellent abrasion resistance and good adhesion to other surfaces. It has a molecular weight ranging between 80,000 and 150,000 g/mol, depending on the material grade, and a Shore hardness between 85A and 95A.

The samples used in this study were produced using 3D printing technology with the FDM (Fused Deposition Modeling) method and three different materials: PLA, PET-G, and TPU. Polymers were supplied by commercial manufacturers and conform to standard specifications for FDM technology. While detailed molecular testing was not performed as part of this investigation, the provided data describe the essential characteristics of the materials used. The thickness of each sample was set at 4 mm and remained consistent across all variants tested. This uniform thickness ensured sufficient stiffness and stability during slip resistance tests and surface roughness measurements. To further eliminate any potential deformations that could affect the flatness of the tested surfaces, we glued each sample to a rigid stabilizing plate using cyanoacrylate adhesive. Future research may include additional molecular analyses to further enhance the understanding of the materials used and explore their influence on surface properties.

The tested materials also exhibit distinct hydrophobic properties, which can influence their behavior under wet conditions. These properties are characterized by the contact angle values reported in the literature for each polymer:

- PLA (Polylactic Acid): PLA demonstrates moderate hydrophobicity, with a contact angle ranging between 65° and 75°. This behavior is attributed to its polar chemical structure, which increases its interaction with water molecules, leading to a relatively lower resistance to moisture [47].
- PET-G (Polyethylene Terephthalate Glycol): PET-G has a higher degree of hydrophobicity compared to PLA, with a contact angle in the range of 80° to 90°. Its less polar molecular structure reduces the water interaction, enhancing its ability to repel moisture. This property can positively impact slip resistance performance under wet conditions [48].
- TPU (Thermoplastic Polyurethane): The TPU exhibits the highest hydrophobicity among the tested materials, with a contact angle ranging from 90° to 100°. Its flexible and non-polar nature results in excellent water repellency, which can contribute to its slip resistance behavior, particularly under wet conditions [49].

These differences in contact angle values highlight the varying levels of hydrophobicity between materials, with TPU being the most water-repellent and PLA the least. Such hydrophobic characteristics are crucial to understanding the materials’ performance in slip resistance tests conducted under dry and wet conditions. Specifically, the higher hydrophobicity of PET-G and TPU suggests their enhanced resistance to moisture, which aligns with their improved slip resistance values observed in wet conditions.

## 3. Results and Discussion

### 3.1. Slip Risk Value Results

**Figure 4:**
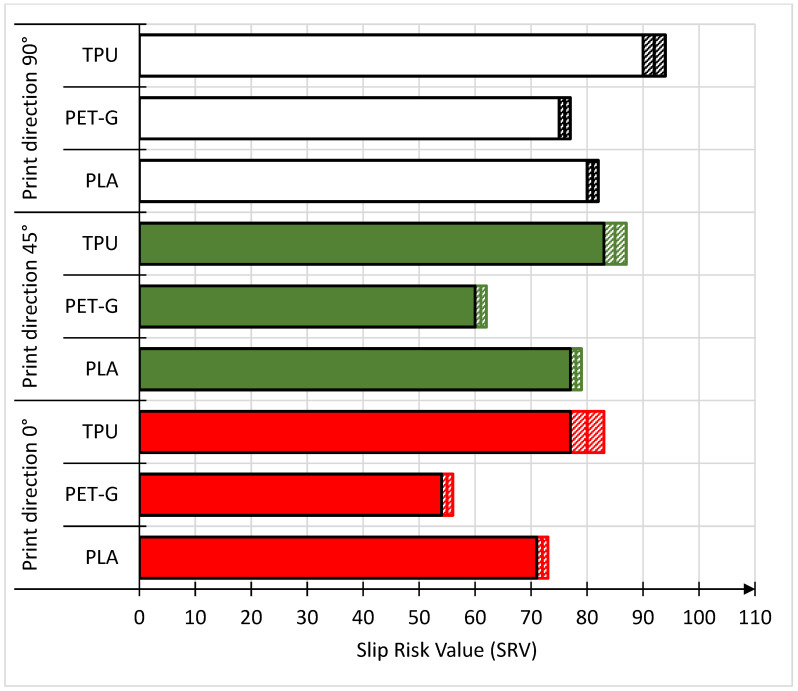
SRVs of the tested samples during the dry test.

**Figure 5:**
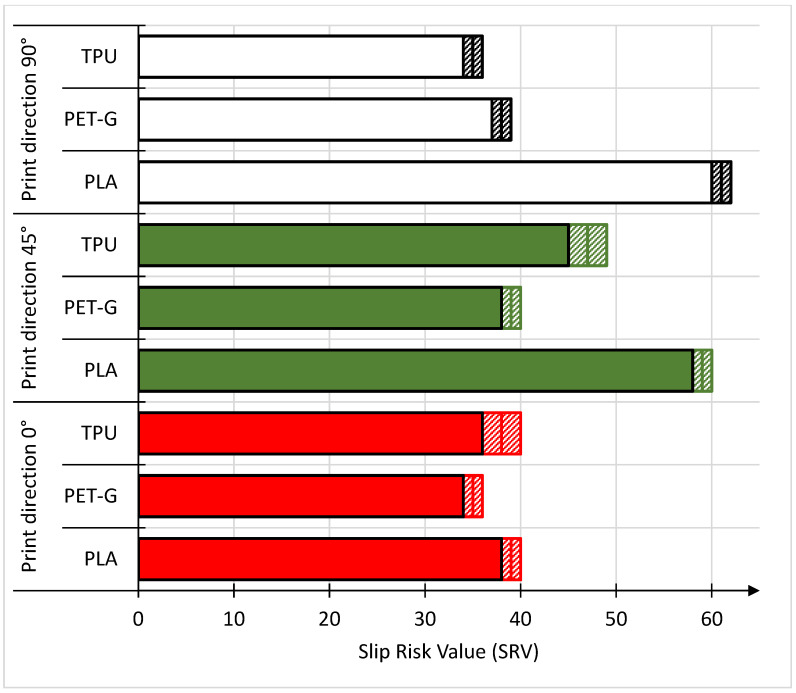
SRVs of the tested samples during the wet test.

Slip risk tests were carried out for the dry test (Figure 4) and the wet test (Figure 5). For each of these tests, three types of material were tested using three different directions of movement of the print head. In the case of the dry measurement test, it was observed that for each type of material, the highest SRVs were obtained for the 90° printing direction. The structure of this direction is similar to that of outdoor ceramic tiles used on stairs with anti-slip grooves, which, according to Waluś in 2022, can reduce slip risk of slips by approximately 22% [3]. When the measured SRVs were applied to the qualitative scale, the samples were classified on this scale as materials with low or extremely low slip risk. This classification allows them to be grouped alongside other floor finishing materials, such as external ceramic tiles with grooves [3], exterior matte tiles [3], floor panels [3], impregnated wood [3], paint for pedestrian crossing lanes, fluorescent coatings [3], industrial aluminum checkered plates [3], roofing materials [3], office floor coverings [3], rubber flooring [3], lacquered wood with matte texture [4], concrete surfaces [50], and oak and beech wood finished with polyurethane or oil coatings [51]. Only two PET-G samples with a PD 90° printing direction and PET-G with a PD 0° printing direction were characterized by a low slip risk. In the further comparative analysis, only the numerical SRV was referred to. In the dry test, the highest SRV was measured for TPU. In the case of this material, the SRV for the print direction (PD) 90° was 92 ± 2, for PD 45° SRV was 85 ± 2 and for PD 0° SRV was 80 ± 3. The lowest SRV was measured for PET-G. For this material, the SRV at PD 90° was 81 ± 1, for PD 45° the SRV 0° was 61 ± 1 and for PD the SRV was 55 ± 1. To sum up the dry test, two factors can be distinguished that affect the SRV. The first is the print direction (PD) and the second is the type of material. In dry slip, greater anti-slip resistance is obtained in the case of unevenness located parallel to the direction of slip. This relationship is common to all tested materials. Further studies were carried out to check whether the direction of printing did not affect the surface roughness, which could translate into the above relationship. In terms of the effect of the type of material on the SRV, the best results are obtained in the case of materials with increased flexibility, as exemplified by the results measured for TPU. The literature mainly includes studies on materials with flexible properties with respect to SRV (Slip Resistance Value) measurements for rubber, which, compared to other materials (mainly brittle ones), did not exhibit significant slip resistance. Under dry conditions, the SRV for rubber was approximately 36, according to the research by Waluś et al. in 2022 [3].

In the case of wet slip testing for TPU and PET-G materials, the direction with the best anti-slip properties was the print direction (PD) 45°. For TPU and PD 45°, the SRV was 47 ± 2, while for PD 90° the lowest SRV was obtained for this material, which was 35 ± 1. In the case of PET-G, the PD 45° direction had an SRV of 39 ± 1. However, the other two print directions had a slight effect on the SRV. For PD 90°, the SRV was 38 ± 1 and for PD 0°, the SRV was 35 ± 1. The only material that maintained the known trend from the dry test, in which the highest values were obtained for PD 90°, was PLA. In the case of this material, the difference in the SRV between the PD 90° and PD 45° directions is small and the values are 61 ± 1 (for PD 90°) and 59 ± 1 (for PD 45°), respectively. However, a large decrease was observed for the PD 0° direction because in this case the SRV was 39 ± 1. It should be noted that, unlike the dry test, in wet slipping conditions, the best anti-slip properties, regardless of the direction, are possessed by the PLA material, which was in second place in the classification in the dry test. When comparing the SRV results for the wet test with the qualitative scale, the PET-G material with the PD 0° direction and the TPU material with the PD 90° direction were classified as materials with a medium risk of slippage. The remaining samples tested were classified on a qualitative scale as materials with a low risk of slipping. In summarizing the results of the wet slip test, it was found that the type of material is of primary importance, while the printing direction (PD) has a small effect on the SRV. However, the best properties are shown by the 45° PD.

### 3.2. Roughness Test Results and Their Analysis

**Figure 6:**
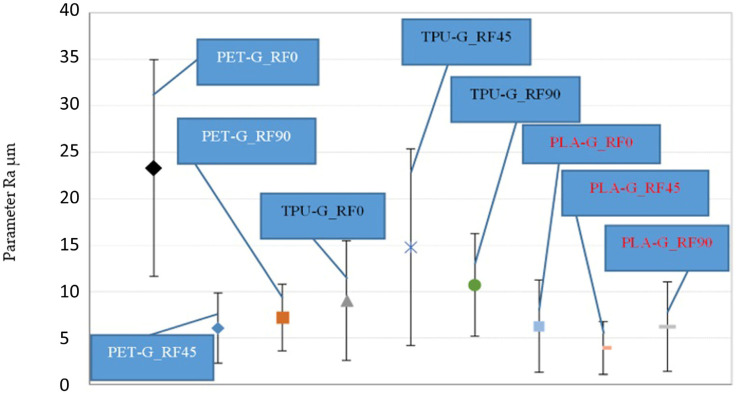
Comparison of the mean value of the Ra roughness parameter along with the scatter of measurement results for the tested PET, TPU, PLA materials, taking into account the printing path direction of 0°, 45°, and 90°. The confidence interval limits for the mean value are given at the significance level of α = 0.1. Where: PET-G_RF0—PET material sample with PD 0° print path application direction; PET-G_RF45—PET material sample with PD 45° print path application direction; PET-G_RF90—PET material sample with PD 90° print path application direction; TPU-G_RF0—PET material sample with PD 0° print path application direction; TPU-G_RF45—PET material sample with PD 45° print path application direction; TPU-G_RF90—PET material sample with PD 90° print path application direction; PLA-G_RF0– PET material sample with PD 0° print path application direction; PLA-G_RF45—PET material sample with PD 45° print path application direction; PLA-G_RF90—PET material sample with PD 90° printing path direction.

By analyzing the changes in the average values of the roughness parameter Ra determined for a given tested material (taking into account the measurements performed parallel and transversely to the direction of the applied printing paths, obtaining the average value of the parameter), it can be stated that for PLA and TPU materials, there is no influence of the direction of the application of the printing paths (with the measurement method described in point 1.3) on the tested roughness parameter Ra, taking into account the scatter of this parameter (Figure 6).

However, in the case of PET material, a significantly higher value of the Ra parameter is observed for the PD 0° printing direction in relation to the PD 45° and PD 90° printing direction of this material. Furthermore, a significantly greater dispersion of the Ra parameter measurement results was observed for the PET material with the PD 0° printing direction in relation to the other printing directions for the PET material (Figure 6), which may indicate surface inhomogeneity, taking into account the surface roughness. Taking into account the average values of the Ra parameter, no changes in this parameter are observed for PET material for the PD 45° printing direction compared to the PD 90° printing direction, taking into account the obtained dispersion of measurement results for the average value at the significance level of α = 0.1. It should also be observed that the dispersion values of the tested Ra parameter for the PET material with the use of the PD 45° and PD 90° printing direction are significantly smaller relative to the dispersion of results for the same material with the use of the PD 0° printing direction. By analyzing the measurement results for Figure 6, comparing the average value of the Raśr parameter taking into account the applied printing directions and the types of materials for a given printing direction, it can be concluded that the PLA material is characterized by the lowest average values of the parameter tested for each of the applied printing directions (for example, when using the PD 0° printing direction, Raśr for the PLA material is 6 ± 5 μm< Raśr for the TPU material where the value is 9 ± 6 μm< Raśr for the PET material where the value is 23 ± 12 μm). The collected results of the roughness measurements defined by the average values of the Ra parameter allow us to state that the lowest value of the tested Ra parameter was achieved for the PLA material using the PD 45° printing direction and was Ra = 4 ± 3 μm, while the highest average value of the Ra parameter was obtained for the PET material using the PD 0° printing direction and was Ra = 23 ± 12 μm.

**Figure 7:**
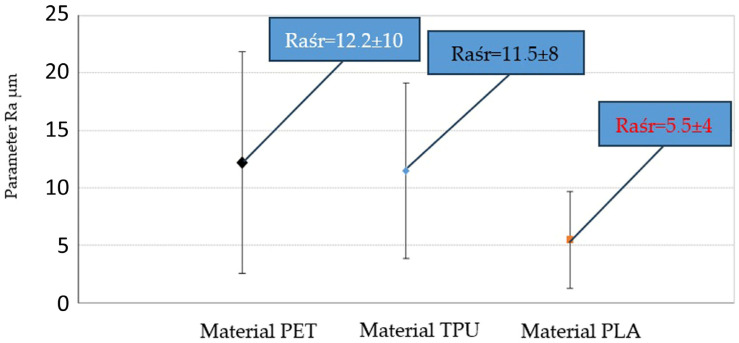
Comparison of the average values of the roughness parameter Ra obtained in the tests of materials (PET, TPU, PLA) after taking into account all the measurement results obtained for a given type of material (the measurement results were averaged taking into account all the applied printing directions PD 0°, 45°, and 90° for a given material). The limits of the confidence intervals for the average value are given at the significance level of α = 0.1.

By comparing the average values of the Ra parameter (Figure 7) for each of the tested materials, taking into account all the results for each applied printing direction of the tested material, it can be stated, taking into account the dispersion of measurement results, that there is no difference in surface roughness (defined by the Ra parameter) between samples made of the tested materials and taking into account all the applied printing directions within a given material. During the surface roughness test, similar relationships were obtained as during the dry slip test, i.e., the highest roughness and the highest SRV coefficient values of the tested samples were obtained for the PD 90° printing direction. This relationship is also maintained for the PLA material during the wet slip test.

### 3.3. Multi-Criteria Statistical Analysis

On the basis of the collected test results, an ANOVA analysis of the significance of independent variables was performed. The dependent variable was the SRV, and the non-dependent variables were: print direction, roughness (Ra parameter), material, and type of test (dry and wet). The first ANOVA analysis was designed to check whether the print direction and roughness of the print have a significant effect on the SRV variable during dry tests (Table 4). The *p*-value for the print direction variable is 0.2048, which is higher than the typical significance level (e.g., 0.05). This means that there is no statistically significant effect of the print direction on the SRV. On the other hand, the *p*-value for roughness is 0.6341, also significantly higher than the 0.05 level, suggesting that roughness does not have a significant effect on the SRV in this sample. The results indicate that the variable print direction and roughness have no statistically significant effect on the dependent variable SRV. The second ANOVA analysis aimed to check whether the print direction and roughness of the variables have a significant effect on the SRV variable during wet tests (Table 5). The results show the same conclusions as those of the dry tests. The *p*-value for the variable print direction is 0.2048, which is higher than the typical significance level (e.g., 0.05). This means that there is no statistically significant effect of the print direction on the SRV. On the other hand, the *p*-value for the variable roughness is 0.6341, also significantly above the 0.05 level, suggesting that the roughness Ra does not have a significant effect on the SRV in this sample. There are no studies in the literature on the effect of the 3D print direction on the SRV, so it is difficult to refer to the results of other researchers. On the other hand, in 2021 Sudoł et al. concluded that surface roughness is not the main determinant of slip resistance, and its final value is influenced by many components that should be considered together and not omitted when designing surface finish [52]. This is consistent with the results of the ANOVA analysis.

The third ANOVA analysis was to test the influence of the type of material used and the type of test (dry and wet) to determine whether these variables have an influence on the variable SRV (Table 6). The study showed that material change has a significant statistical influence on the variable SRV (the *p*-value was 0.0044) and the type of test also had a significant statistical influence on the variable SRV (the *p*-value was <0.0001). The interaction between variable material and the type of test is also significant (*p* = 0.0265), which means that the influence of material on SRV varies depending on the type of test. The results indicate that both the material, the type of test, and their interaction have a statistically significant influence on the dependent variable “SRV”.

## 4. Conclusions

This study investigated the slip of surfaces manufactured using 3D printing technology, focusing on three materials: PLA, PET-G, and TPU—under dry and wet conditions. The research aimed to determine how material type, printing direction, and surface roughness influence slip resistance, measured using the Slip Resistance Value (SRV). The primary research question addressed how these factors interact and their relative importance in reducing slip risk. The results demonstrated that TPU exhibited the highest SRV under dry conditions, while PLA performed best in wet environments. PET-G consistently showed lower SRV compared to TPU and PLA. Regarding printing direction, the direction of printing, the 90° configuration provided the highest slip resistance in dry conditions, whereas the 45° configuration was more effective for TPU and PET-G in wet conditions. Surface roughness and printing direction did not significantly influence SRV, as determined by statistical analysis. However, material choice and environmental conditions (dry vs. wet) emerged as the most critical factors. This study makes a valuable contribution to the literature by offering new insights into the interplay of material properties, printing parameters, and environmental factors in determining slip resistance. It underscores the potential of 3D printing to design customized surfaces with tailored anti-slip properties, advancing the broader understanding of surface engineering for safety applications. Despite its contributions, the study has some limitations. It focused on three specific materials and printing parameters, limiting the generalizability of the findings to other materials or 3D printing methods. Additionally, the roughness measurements were based on a limited range of profilometry techniques, which may not fully capture the surface characteristics affecting slip resistance. External factors, such as long-term wear or surface contamination, were also not considered. Future research should expand the scope by including additional 3D printing materials and exploring advanced post-processing methods to enhance surface properties. Investigations into the effects of wear, contamination, and long-term use on slip resistance are also recommended. Furthermore, exploring the relationship between microstructural characteristics and SRV using advanced imaging techniques could provide deeper insights. Developing predictive models for slip resistance based on material, environmental, and manufacturing parameters would further optimize surface design. By addressing these limitations and building on the current findings, future studies can continue to advance the application of 3D printing technology in designing surfaces with enhanced safety properties for diverse environments.

## Figures and Tables

**Table 1:** Qualitative scale of slipping risk depending on the SRV.

Risk of Slipping	SRV
High	≤25
Moderate	25–35
Low	35–65
Extremely Low	≥65

**Table 2:** Photos of the 9 printed samples tested were taken with a Bresser BIOLUX ICD BINO 80× LED optical microscope.

PLA		
0°	45°	90°
** 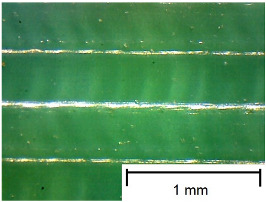 **	** 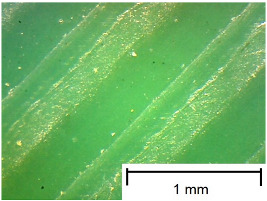 **	** 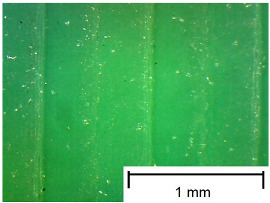 **
PET-G		
0°	45°	90°
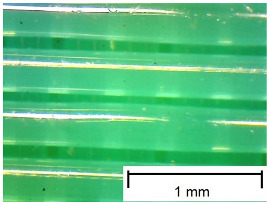	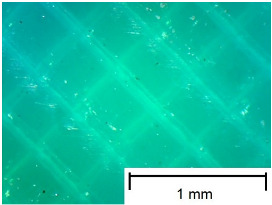	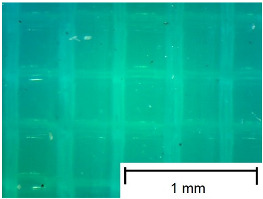
	TPU	
0°	45°	90°
** 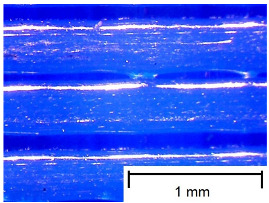 **	** 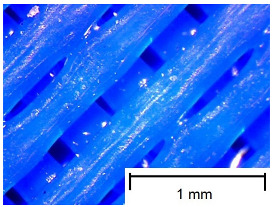 **	** 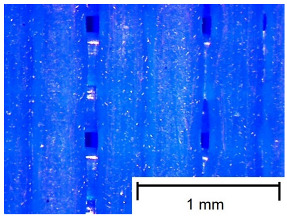 **

Thirty-fold magnification was used.

**Table 3:** Detailed parameters set during printing of the research plates.

	Type of Material		
3D Printing Parameters	PLA	PET-G	TPU
Nozzle Temperature [°C]	215	230	228
Bed Temperature [°C]	60	80	45
Infill [%]	85	85	85
Layer Thickness [mm]	0.2	0.2	0.2
Print Speed [mm/s]	60	60	25
Print Cooling [%]	100	0	50
Wall Thickness [mm]	0.8	0.8	0.8
Infill Type	straight lines	straight lines	straight lines

**Table 4:** ANOVA analysis for the dependent variable SRV versus the independent variables print direction and roughness during dry testing, where df—degrees of freedom, F—Fisher F statistic.

Factor	Sum of Squares	df	F	*p*-Value
Print direction	213.52	1	2.02	0.2048
Roughness	26.53	1	0.25	0.6341
Residual	633.47	6		

**Table 5:** ANOVA analysis for the dependent variable SRV versus the independent variables print direction and roughness during wet testing, where df—degrees of freedom, F—Fisher F statistic.

Factor	Sum of Squares	df	F	*p*-Value
Print direction	0.74	1	0.0063	0.9391
Roughness	141.05	1	1.2082	0.3138
Residual	700.50	6		

**Table 6:** ANOVA analysis for the dependent variable SRV versus the independent variables material, type of study, material and type of research during dry and wet testing, where df—degrees of freedom, F—Fisher F statistic.

Factor	Sum of Squares	df	F	*p*-Value
Material	810.78	2	8.83	0.0044
Type of study	4355.56	1	94.92	<0.0001
Material and type of research	458.11	2	4.99	0.0265
Residual	550.67	12		

## Data Availability

The original contributions presented in this study are included in the article. Further inquiries can be directed to the corresponding author.

## Abbreviations

The following abbreviations are used in this manuscript:

PLA	Polylactic Acid
PET-G	Polyethylene Terephthalate Glycol
TPU	Thermoplastic Polyurethane
BSRT	British Portable Skid Resistance Tester
BPN	British Pendulum Number
RSV	Slip Resistance Value
FDM	Fused Deposition Modeling
SLA	Stereolitography
PD	Print direction
df	degrees of freedom
